# Cyclic fatigue of a repaired 4 YSZ ceramic: Effect of the repair protocol on the adhesive and mechanical behavior

**DOI:** 10.1016/j.heliyon.2023.e23709

**Published:** 2023-12-15

**Authors:** Pablo Machado Soares, Lucas Saldanha da Rosa, Rafaela Oliveira Pilecco, Gabriel Kalil Rocha Pereira, Amanda Maria de Oliveira Dal Piva, João Paulo Mendes Tribst, Luiz Felipe Valandro, Cornelis Johannes Kleverlaan, Marilia Pivetta Rippe

**Affiliations:** a Post-Graduate Program in Oral Sciences, Center for Development of Advanced Materials, Division of Prosthodontics-Biomaterials, Federal University of Santa Maria (UFSM), Santa Maria, Brazil; b Department of Dental Materials Science, Academic Centre for Dentistry Amsterdam (ACTA), Universiteit van Amsterdam and Vrije Universiteit, Amsterdam, North Holland, the Netherlands; c Department of Reconstructive Oral Care, Academic Centre for Dentistry Amsterdam (ACTA), Universiteit van Amsterdam and Vrije Universiteit, Amsterdam, North Holland, the Netherlands

## Abstract

**Objective:**

To evaluate the effect of different surface treatments on the morphology, shear bond, and flexural fatigue strength of a repaired translucent zirconia.

**Methods:**

Monolithic disc-shaped specimens of translucent zirconia were prepared and ground to simulate repair areas. Four groups underwent different treatments: Air-MDP (air-abrasion with alumina particles and 10-MDP primer), *Si*-Sil (silica-coated alumina particles with MDP-containing silane), *Si*-MDP (silica coating with 10-MDP primer), and Uni adhe (universal adhesive). After roughness measurements and treatments, repairs were done using resin composite. Shear bond and flexural (n = 15) fatigue tests were performed. Surface topography, interfacial analysis, fractographic, and finite element analysis were conducted.

**Results:**

The zirconia roughness was similar between the groups, however, the surface topography was modified according to the surface treatments. *Si*-Sil generated higher and more stable bond strength values (20.69 MPa) between translucent zirconia and resin composite when compared to Uni adhe (15.75 MPa) considering the fatigue bond strength scenario, while it was similar to *Si*-MDP (17.70 MPa) and Air-MDP (18.97 MPa). Regarding the mechanical behavior, *Si*-Sil (680.83 MPa) also showed higher and significantly different fatigue strength when compared to Uni adhe (584.55 MPa), while both were similar to *Si*-MDP (634.22 MPa) and Air-MDP (641.86 MPa).

**Conclusion:**

The association of mechanical and chemical approaches is essential for long-term bond strength and optimized mechanical behavior, being air-abrasion protocols and the use of silane and/or MDP-based primers suitable for zirconia repair protocols. It was found that relying solely on a universal adhesive was not as effective as other options available.

**Clinical significance:**

The surface treatment of repair protocols affects translucent zirconia's morphology. To enhance fatigue behavior in repaired monolithic zirconia, air abrasion is crucial. Exclusive use of a universal adhesive is less effective than other choices. A primer containing silane/MDP holds the potential for stable bond strength and optimized mechanical performance.

## 1 Introduction

In clinical practice, dental ceramics are commonly used for prosthetic rehabilitation. This is because they are biocompatible, exhibit excellent mechanical behavior, and allow for more conservative tooth preparations compared to traditional metal-ceramic crowns [1]. In this context, monolithic zirconia crowns emerged as one of the most eminent restorative options to overcome the incidence of chipping [2], due to the higher mechanical strength when compared to bilayer systems [3].

As a polycrystalline material, yttrium stabilized zirconia (YSZ) presents the highest mechanical strength among the available dental ceramics [2,4,5], even when considering high translucent variations (4 YSZ and 5 YSZ). These new generations present an increased percentage of yttrium stabilizer and also a cubic phase, which made this material more translucent and esthetic, since it provides better light interaction compared to previous generations of zirconia (3 YSZ), thus being indicated for full-crown monolithic restorations, for both anterior and posterior regions [2].

Despite these advantages, 4 YSZ presents a high elastic modulus (200 GPa) when compared to the tooth structure (i.e. dentin – 18 GPa), which may result in a problem when associated with its brittleness and suitability to slow crack growth [6]. Hence, even though they present high levels of clinical performance (98 % survival rate in 5 years) [7], technical complications such as fractures have also been reported for monolithic zirconia crowns [8,9]. When these kinds of failures occur, direct intra-oral repair with resin composite may consist of an effective and simple alternative for clinicians, since it is less time-consuming, cheaper than the restoration replacement, and can be done in a single session [10,11]. Besides, it also post-pones more invasive interventions that would intensify tooth remnant removal, delaying the consequences of a repetitive restorative cycle, and increasing the longevity of teeth [12]. Although, there are still gaps in the best protocol to perform repairs using polymeric materials, mainly when considering the repair of highly translucent polycrystalline materials.

Zirconia ceramics are known for their challenged bonding affinity when compared to glass ceramics, due to the absence of glass content in its microstructure and chemical inertness [2,13]. For an adequate bond strength between zirconia and resin composite, mechanical interlocking and chemical mechanisms are essential [14]. Therefore, several surface treatments and bonding agents have been evaluated for zirconia restorations before the repair [15]. Currently, the most usual surface treatment is air-abrasion with alumina particles, associated with the application of a 10- Methacryloyloxydecyl Dihydrogen Phosphate (10-MDP) primer, which increases the ceramic surface roughness, wettability, and surface energy, besides the chemical bonding achievement [[14], [15], [16], [17], [18]].

Recently, other studies evaluated alternative protocols to increase the chemical bonding, such as air-abrasion with silica-coated alumina particles and the use of a silane agent, which also has been showing great performance [19]. Although air-abrasion protocols can be useful, they may also cause surface damage which could compromise the mechanical performance of ceramics. This is due to the introduction of defects that can act as trigger points for stress concentration and crack initiation [20,21]. Thus, the use of different parameters during air-abrasions, such as time, pressure, and grain-size particles [22,23]; the isolated use of a 10-MDP primer [24], or even, more recently, the use of universal adhesives [25], have been explored. Despite this, there is still a lack of scientific evidence to support a gold standard protocol for directly repairing zirconia, particularly the more translucent ones. Therefore, further studies are needed to define such a protocol to answer the following question: which intraoral zirconia surface condition and bonding agent are indicated when requiring a clinical repair approach?

Considering the aforementioned factors, this study aimed to evaluate the effect of different zirconia surface treatments and adhesive systems on the morphology, shear bond strength, and mechanical fatigue behavior of a resin composite-repaired 4 YSZ ceramic. The null hypotheses consisted that different zirconia surface treatments would not affect 1) ceramic micromorphology, 2) static and fatigue shear bond strength, and 3) fatigue biaxial flexural strength.

## 2 Materials and methods

### 2.1 Study design

The materials used in the present study, commercial names, batch number, and composition are depicted in Table 1 and the investigated groups are described in Table 2.

**Table 1:** Materials used in the present study, commercial names, manufacturers, batch number, and main composition.

Material	Commercial Name	Manufacturer (Batch number)	Main composition
**Yttria-stabilized tetragonal zirconia polycrystal (4YSZ)**	IPS e.max ZirCAD MT	Ivoclar AG (V26180)	ZrO_2_; 8 % weight of Y_2_O_3_; HfO_2_; Al_2_O_3_; other oxides.
**Nanohybrid resin composite**	Tetric EvoCeram	Ivoclar AG (Z03XMX)	Urethane dimethacrylate 5 < 10 %; Bis-GMA 3–7%; ytterbium trifluoride 3–5%; ethoxylated bisphenol A dimethacrylate 3–5%
**Primer MDP**	Alloy Primer	Kuraray Noritake Dental Inc. -(210,121)	Acetone; 10-Methacryloyloxydecyl dihydrogen phosphate (MDP); 6-(4-Vinyl-benzyl-*N*-propyl) amino-1,3,5-triazine-2,4-dithione
**MDP-containing silane**	Monobond N	Ivoclar AG (Z02M0Y)	Alcohol solution of silane methacrylate; phosphoric acid methacrylate (MDP); sulphide methacrylate
**Universal adhesive system**	Adhese Universal	Ivoclar AG (Z047S9)	MDP, MCAP, HEMA, BisGMA, D3MA; Water; Ethanol 25.0 %; Highly dispersed silicon dioxide 4.0 %; Initiators and Stabilisers 4.0 %
**Aluminum oxide**	White aluminum oxide 50 μm	Zest Dental Solutions (L2BN5)	50 μm Al_2_O_3_
**Silica-coated aluminum oxide**	Cojet Sand	3 M ESPE (9536662)	Aluminum oxide 30 μm, amorphous silica

**Table 2:** Study design.

Groups	4 YSZ surface treatments	Repair material	Tests to be performed
**Air-MDP**	Air-abrasion with alumina particles (50 μm) at 10 mm of distance and 2 bar of pressure for 10 s + Primer MDP (10 s)	Nanohybrid resin composite (Tetric EvoCeram, Ivoclar)	Shear bond strength test (n = 10) Fatigue shear bond strength test (n = 15) Fatigue Biaxial flexural strength test (n = 15) Surface roughness (n = 15) Surface topography (n = 1) Interface analysis (n = 1) Fractographic analysis (n = 1) Finite element analysis (n = 1)
***Si*-Sil**	Silica-coated alumina particles air-abrasion (30 μm) at 10 mm of distance and 2 bar of pressure for 10 s + MDP-containing silane (60 s)		
***Si*-MDP**	Silica-coated alumina particles air-abrasion (30 μm) at 10 mm of distance and 2 bar of pressure for 10 s + Primer MDP (10 s)		
**Uni Adhe**	Universal adhesive (active application for 20 s)		

### 2.2 Sample size calculation

The sample size was calculated for each method using a statistical software program (OpenEpi) and the data from a pilot study evaluating each group for biaxial flexural strength (n = 5) and shear bond strength (n = 5) tests (95 % confidence interval and a statistical power of 80 %). However, for the static tests, 10 specimens were used, and 15 specimens for all fatigue tests, which is also by previous studies using similar test geometries [5,26,27].

### 2.3 Fatigue shear bond strength

#### 2.3.1 Specimen's preparation

A disc of 4 YSZ (IPS e. max ZirCAD MT, Ivoclar AG, Schaan, Liechtenstein) was cut in blocks (10 × 10 × 16 mm considering the sintering shrinkage) with a diamond disc coupled to a hand-piece and an electric motor (Perfecta LA 623 T, 1000 to 40,000 rpm – W&H, Bürmoos, Austria). The blocks were attached to a metal washer to be grounded in a polishing machine (EcoMet/AutoMet 250, Buehler, Lake Bluff, USA) until the achievement of a cylindric shape (Ø = 8 mm). Then, discs were obtained by slicing the cylinders using a precision cutting machine (IsoMet 1000, Buehler) under constant water cooling. After, the discs were polished with silicon carbide (SiC) sandpapers #600- and #1200-grit (3 M, Sumaré, Brazil). The specimens were cleaned and sintered (Zyrcomat 6000 MS, VITA Zahnfabrik, Bad Sackingen, Germany) according to the manufacturer's recommendations (heating rate 10 °C/min until 900 °C; holding phase at 900 °C for 30 min; heating rate 3 °C/min until 1500 °C; holding phase at 1500 °C for 120 min; cooling rate 8 °C/min). The final dimensions of the discs were: Ø = 6 mm, 1.5 mm of thickness).

#### 2.3.2 Surface treatments and roughness analysis

For all specimens, a grinding protocol was performed using a 4219 F diamond bur (grain size of 46 μm, KG Sorensen, Cotia, São Paulo, Brazil) coupled to a multiplier contra-angle (T2 REVO R170 contra-angle handpiece up to 170,000 rpm, Sirona, Bensheim, Germany) parallel to the bonding surface of the discs to mimic a roughened surface before the repair in a clinical scenario. For this, a permanent mark was made on the specimen's surface for standardization. Then, grinding was done under water-cooling with light digital pressure and oscillatory movements until the mark was removed [28].

After the grinding protocol, the specimens were cleaned in an ultrasonic bath for 5 min with alcohol and randomly assigned to the experimental groups according to the surface treatments and bonding agents, as described in Table 2, with the Air-MDP group being adopted as a control group. The surface roughness analysis using a profilometer (Mitutoyo SJ-410, Mitutoyo Corporation, Kawasaki, Japan) through six measurements for each specimen at different points considering the parameters Ra and Rz (cut 5; λC 0.8 mm; λS 2.5 μm), by ISO 4287: 1997 [29]. Ra is the arithmetical mean of the absolute values of peaks and valleys measured from a mean plane (μm), and Rz is the average distance between the five highest peaks and five major valleys of a surface (μm). A One-way ANOVA statistical test was performed and no statistical difference was found between the groups (p > 0.05), thus assuring similar surface conditions before the surface treatments. The groups that received air-abrasion protocols (Table 2) were submitted to a new surface roughness analysis as described above (n = 15).

#### 2.3.3 Static and fatigue shear bond strength test

**Fig. 1:**
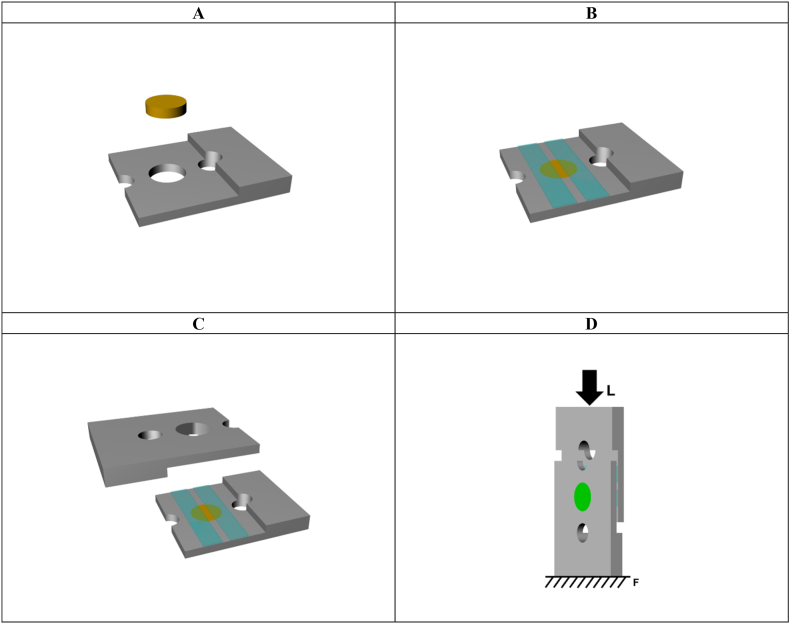
Specimen preparation for the shear bond strength test. The zirconia discs were coupled to a metallic device (A), and two tapes were positioned over the disc to reduce the bonding interface to 1 mm (B). A second metallic device was fixed over the first one (C) and then the resin composite was applied over the zirconia disc. The specimen was fixed vertically in the machine, and a load was applied over the metallic device, which acted on the specimen until the failure.

After surface roughness analysis, the bonding agents (primer MDP, MDP-containing silane, or universal adhesive) were applied over the treated zirconia surface, according to the study design (Table 2) and following the manufacturer's recommendations. Each 4 YSZ specimen was positioned in a metallic device for the shear bond strength test (Fig. 1A). To reduce the bonding area between zirconia and resin composite, adhesive tapes (Scotch Magic Tape, 3 M, Saint Paul, USA) were positioned 1 mm from each other over the 4 YSZ (Fig. 1B), by the use of a digital caliper (Absolute digimatic, Mitutoyo, Kawasaki, Japan) [27]. The metallic pair device was positioned over the first one in a standard position (Fig. 1C), through a polyvinyl siloxane matrix (Express XT Putty, 3 M ESPE, Seefeld, Germany), and the nanohybrid resin composite (Tetric EvoCeram, Ivoclar AG) was inserted over the ceramic (Ø = 6 mm and 1.5 mm thickness) in one increment and light cured (Radii-cal LED curing light, SDI, Bayswater, Australia) for 20 s (Fig. 1D).

To determine the fatigue load profile, a static shear bond strength test was performed (n = 10) after a period of storage at 37 °C for at least 24 hours. The specimens were tested in a universal testing machine (Instron 6022; Instron, Norwood, USA) at a crosshead speed of 0.5 mm/min. The metallic devices were positioned vertically in a metal base, and the load was applied over the metallic device (which acted on the specimen) through a flat stainless-steel load applicator (Ø = 10 mm) and a 1 KN load cell connected to the machine (Fig. 1D). The loads for bonding failure data were recorded and the shear strength “S” (in MPa) was calculated according to equation (S = L/A); where “L” is the load to failure (N) and “A” is the cross-sectional area of the interface, that was calculated for each specimen considering the bonding interface dimensions (overall mean = 5.8 mm^2^).

For the fatigue test using the same shear bond strength device as described above, the specimens (n = 15) were tested through a cyclic fatigue methodology [27] in an adapted equipment (ACTA, Amsterdam, Netherlands) that acts using a pneumatic system. Cyclic loads were applied through a hexagon stainless-steel piston (Ø = 13 mm), initiating with a 10 N load for 10,000 cycles at the ceramic-resin interface. Incremental steps of 10 N/10,000 cycles were applied with 3 Hz of frequency until failure (Fig. 1). The fatigue failure load (FFL) data (N) and the number of cycles for failure (CFF) were recorded and the fatigue shear bond strength (MPa) was calculated according to the static test.

#### 2.3.4 Failure analysis

All tested specimens were analyzed under a stereomicroscope (Discovery V20, Carl Zeiss, Gottingen, Germany) at a magnification of 15 × , with failures categorized as predominantly adhesive (more than 50 % of adhesive failure) at the ceramic/resin interface; and predominantly cohesive (more than 50 % cohesive failure) within the ceramic or resin composite [27]. Representative specimens of each group were evaluated under scanning electron microscopy (SEM, Evo LS15, Carl Zeiss, Gottingen, Germany) at a magnification of 100 × , to obtain images of each type of failure.

### 2.4 Fatigue biaxial flexural strength

#### 2.4.1 Specimen's preparation

For the biaxial flexural strength test, 4 YSZ discs (Ø = 15 mm, 1 mm thickness) were obtained, polished, submitted to the same grinding protocol, and sintered, as previously described [5,28,30]. All specimens were cleaned and distributed among the different groups, followed by the aforementioned surface treatments (Table 2).

The resin composite was applied with incremental technique on the zirconia discs through a polyvinyl siloxane matrix (0.4 mm thickness). After application, a smooth glass plate was pressed against the resin composite, and the specimens were light-cured at 1200 mW/cm^2^ (Radii-cal LED curing light, SDI, Bayswater, Australia) for 60 s. The top surface of the resin composite was subsequently polished with SiC sandpaper #600- and # 1200-grit until a surface free of defects was obtained in the desired final thickness (0.4 mm – resin composite, 1 mm - ceramic; total thickness = 1.4 mm). The thickness was meticulously checked with a digital caliper (Absolute digimatic, Mitutoyo, Kawasaki, Japan), being any specimen with thickness out of the range of 1.4 mm ± 0.02 was replaced by a new one to avoid the effect of thickness factor on the stress concentration during the mechanical test. The specimens were stored in distilled water for at least 24 hours at 37 °C before testing.

#### 2.4.2 Fatigue biaxial flexural strength test

The fatigue test was performed in a fatigue testing machine (ACTA, Amsterdam, Netherlands), by applying loads through a pneumatic system, with a steel piston (Ø = 1.6 mm) positioned at the center of the top surface of each specimen (resin composite layer facing up). The restorative set was immersed in distilled water, and positioned on a base of 3 spheres equidistant by 10 mm [5]. An adhesive tape was positioned over the compression area for better stress distribution and to make fragment dispersion difficult after fracture. The test was performed using the cyclic fatigue methodology [21], starting with an initial load of 100 N (10,000 cycles), followed by loads of 150 N, 175 N 200 N, 225 N (step size of 25 N), and so on (10,000 cycles/step and with 1.4 Hz of frequency) until the specimen fracture. The inducted stress (MPa) was determined through the finite element analysis as described below in topic 2.6.

#### 2.4.3 Failure and interface analysis

The fractographic analysis of the failed specimens were analyzed under a stereomicroscope (Discovery V20, Carl Zeiss, Gottingen, Germany), and one representative specimen from each group was analyzed under SEM at 150 × and 500 × magnifications to evaluate the interface between 4 YSZ and resin composite, besides to determine failure origin.

### 2.5 Surface analysis

SEM analysis was also performed to determine the topographic pattern of the 4 YSZ after the surface treatments and conditioning protocols. For this, two additional specimens from each group were made and coated with a gold-palladium alloy and then analyzed with 5000 × magnifications.

### 2.6 Finite element analysis (FEA)

To determine the maximal principal stress (MPa) on the center of the repaired zirconia specimens during the biaxial flexural strength test, a three-dimensional (3D) FEA was carried out. Models of the zirconia discs, resin composite, and piston were obtained considering the *in vitro* test assembly (Rhinoceros, version 5.0 SR8, McNeel North America). The Young's modulus (E) and Poisson ratio (v) of the evaluated materials (4 YSZ - E = 200 GPa; v = 0.31; resin composite - E = 12 GPa; v = 0.24; stainless-steel ring/sphere - E = 190 GPa; v = 0.27), were obtained from previous studies and used during the analysis [26,31]. The FFL data (in N) of each specimen were used to generate an individual simulation and to obtain strength results in megapascal (MPa) [32], using the 10 % mesh convergence test to assure the quality of the results and depict differences associated with the factor under study (significant when beyond 10 %) [33]. To perform the structural analysis by the fatigue tests, the materials were considered isotropic, linear, and homogeneous. The data analysis was performed using a computer-aided engineering software program (ANSYS 19, ANSYS Inc., Houston, USA). Finally, the obtained strength data were evaluated qualitatively.

### 2.7 Statistical analysis

The data were subjected to normality (Shapiro-Wilk) and homoscedasticity (Levene) tests to determine the most appropriate statistical analysis for each factor. Since the data assumed a normal distribution, One-Way Analysis of Variance (ANOVA) and Tukey (post hoc) tests were performed to evaluate the surface treatment system factor for roughness, static shear bond strength test, and fatigue tests.

Kaplan Meier test with Mantel-Cox post-hoc (log-rank) was performed for the survival rate (α = 0.05) of the shear and biaxial fatigued data, using the SPSS statistical program in its version 21 (IBM, Chicago, USA).

## 3 Results

**Fig. 2:**
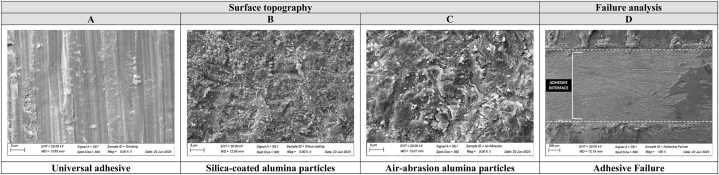
SEM images depicting the surface topography for each group (A, B and C). Representative SEM image of a predominantly adhesive failure at the interface between zirconia and resin composite after the fatigue shear bond strength test (D), which represents the failure pattern for all evaluated groups (100 % of adhesive failures).

No statistical difference was observed among the groups regarding the surface roughness (Table 3) analysis (Ra: p = 0.139, F = 2.066; Rz: p = 0.399, F = 0.940). The surface analysis by SEM showed that the air-abrasion with alumina and silica-coated alumina particles (Air-MDP, *Si*-Sil, *Si*-MDP) depicted a smoother and more regular surface (Fig. 2A–D), with shallower defects (Fig. 2B and C), while a more irregular and scratched surface topography was observed in the just grounded specimens (Fig. 2A).

**Table 3:** Mean (standard deviation) of the roughness analysis. Mean (standard deviation) for the static shear bond strength test. Mean (95 % confidence interval) of the fatigue strength (MPa) and cycles for failure (CFF) for fatigue shear bond and flexural strength tests.

Groups	Roughness analysis		Static Shear test (MPa)	Fatigue shear bond strength test		Fatigue biaxial flexural strength test		
	Ra	Rz		Bond Strength (MPa)	Cycles for failure (CFF)	Load for failure (FFL)	Cycles for failure (CFF)	Fatigue strength (MPa)^a^
**Air-MDP**	0.948 (0.092)^A^	5.921 (0.499)^A^	14.8 (10.29)^B^	19.0 (16.83–21.10)^AB^	103,702 (92,435–114,968)^A^	280 (263–297)^AB^	56,811 (50,429–63,192)^AB^	642^AB^
***Si*-Sil**	1.013 (0.131)^A^	6.143 (0.633)^A^	40.1 (7.44)^A^	20.7 (18.55–22.83)^A^	113,759 (100,653–126,865)^A^	297 (278–316)^A^	61,225 (54,101–68,349)^A^	681^A^
***Si*-MDP**			13.5 (10.11)^B^	17.7 (15.03–20.37)^AB^	94,836 (79,762–109,911)^A^	277 (258–295)^AB^	54,534 (47,288–61,779)^AB^	634^AB^
**Uni Adhe**	0.928 (0.130)^A^	5.894 (0.491)^A^	36.1 (11.13)^A^	15.8 (13.07–18.42)^B^	86,017 (69,752–102,282)^A^	255 (234–276)^B^	43,935 (35,965–51,906)^B^	585^B^

Capital letters show significant differences between the groups for each analysis (columns) depicted by One-Way ANOVA (roughness and static shear bond strength test) and Kaplan Meier and log-rank (biaxial and shear fatigue tests).

a The differences in fatigue strength between the groups were considered significant when higher than 10 % considering the mesh convergence.

**Fig. 3:**
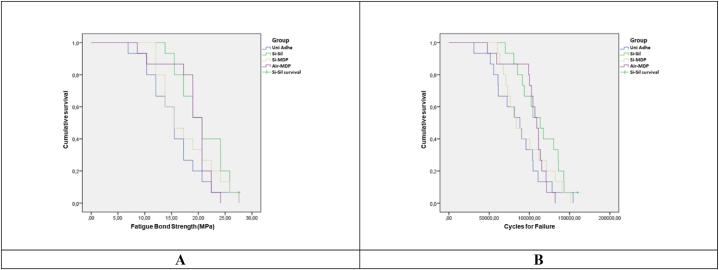
Survival graphs obtained in the Kaplan-Meier and Mantel-Cox (log-rank) post hoc tests, indicating the probability of the specimens of each group to exceed the respective fatigue strength (A) and number of cycles for failure (B) step without failure during the fatigue shear bond strength test.

Shear bond strength tests, as well as the biaxial fatigue test results, are depicted in Table 3, Table 4, and Fig. 3. The static shear bond strength was affected by the zirconia surface treatment (p < 0.001; F = 20.020). The air-abrasion with alumina particles (Air-MDP) and silica coating associated with MDP-primer (*Si*-MDP) depicted lower values of static bond strength when compared to the use of universal adhesive (Uni Adhe) and silica coating associated with MDP-containing silane (*Si*-Sil) were applied.

**Table 4:** Percentage of survival rates (standard error) obtained in the Kaplan-Meier survival test, indicating the probability of the specimens of each group to exceed the respective fatigue failure load (FFL) and number of cycles for failure (CFF) step without failure during the fatigue biaxial flexural strength, and its respective standard error values.

Groups	FFL (N)/CFF										
	150/10,000	175/20,000	200/30,000	225/40,000	250/50,000	275/60,000	300/70,000	325/80,000	350/90,000	375/100,000	400/110,000
**Air-MDP**	100	100	100	93 (64)	67 (12)	33 (12)	20 (10)	7 (6)	0	–	–
***Si*-Sil**	100	100	100	100	87 (9)	47 (13)	27 (11)	20 (10)	7 (6)	0	–
***Si*-MDP**	100	100	93 (64)	87 (9)	67 (12)	40 (13)	20 (10)	0	–	–	–
**Uni Adhe**	100	93 (64)	87 (9)	67 (12)	40 (13)	20 (10)	13 (9)	0	–	–	–

* The sign ‘-’ indicates the absence of a specimen of such condition being tested at this respective step.

One-way ANOVA showed that the surface treatment also affected the fatigue bond strength of the repaired zirconia (p = 0.048; F = 2.810). *Si*-Sil depicted higher values for fatigue strength, compared to the Uni Adhe group, however, both (*Si*-Sil and Uni Adhe) were similar to the *Si*-MDP and Air-MDP groups (p ≥ 0.05) (Fig. 3A). Besides, all the failures were adhesive (100 %), both for static and fatigue shear bond strength tests (Fig. 2D).

Regarding flexural strength, one-way ANOVA showed that the zirconia surface treatment affected the mechanical behavior of the set (p = 0.036; F = 3.045). The *Si*-Sil group presented the highest values of FFL and CFF, followed by *Si*-MDP and Air-MDP, however without statistically significant differences (p ≥ 0.05). Uni Adhe depicted again the lowest values of FFL and CFF, being statistically different from *Si*-Sil (p < 0.05). The same results (significant difference between *Si*-Sil and Uni Adhe) can be observed considering the mesh convergence for the fatigue strength data (MPa). The survival rates corroborated such findings since the Uni Adhe started to fail before the other groups (Fig. 3B). Also, when a load of 250 N was applied during the fatigue test, *Si*-Sil had an 87 % survival probability, while Uni Adhe had just 40 % at the same step (Table 4).

**Fig. 4:**
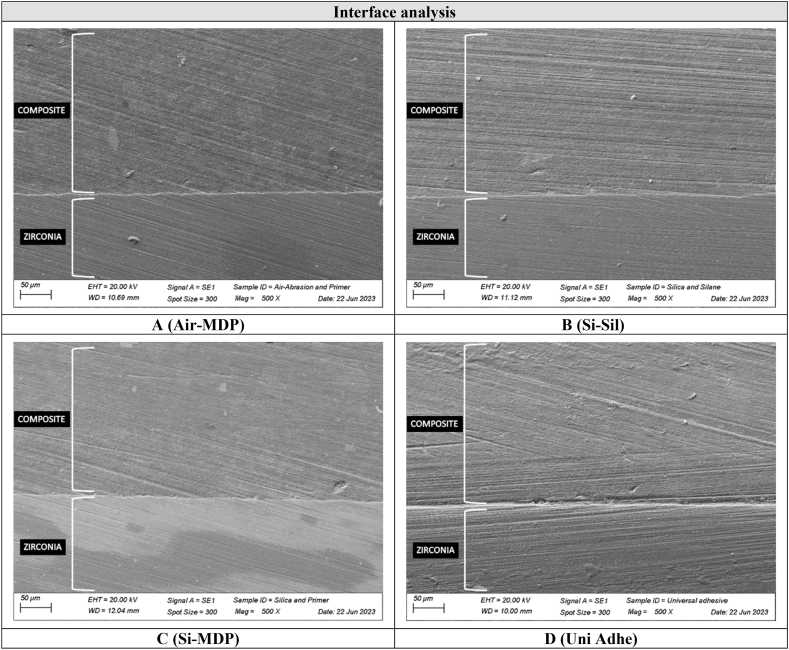
SEM images of the interface between zirconia and resin composite for each group. A thicker and more irregular layer can be seen for the Uni Adhe group (D) when compared to the air-abraded groups (A, B and C), where the universal adhesive takes place over the ground zirconia.

**Fig. 5:**
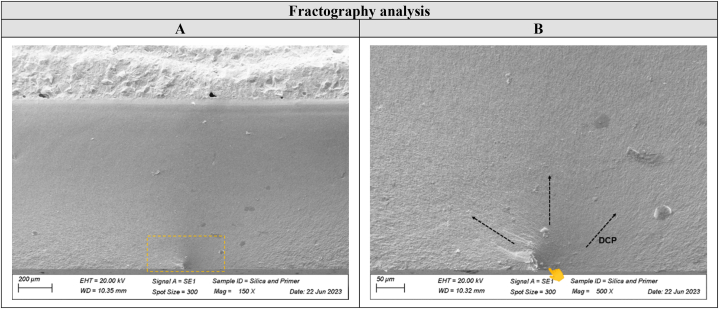
SEM images of the fractographic analysis for a representative specimen. The yellow square (A) indicates the failure origin region in a lower magnification (150×). The yellow pointer indicates the failure origin (B) in a higher magnification (500×), while the black arrows indicate the direction of crack propagation (DCP). (For interpretation of the references to colour in this figure legend, the reader is referred to the Web version of this article.)

**Fig. 6:**
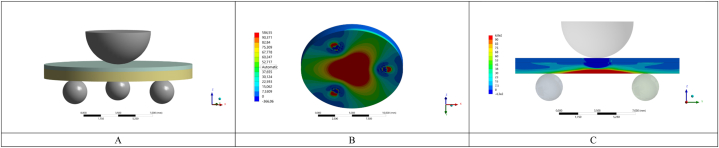
Flexural biaxial fatigue test by the piston on three balls geometry, with the resin composite facing up (A). Representative images of the finite element analysis (FEA), depicting the stress distribution over the zirconia disc under tensile in different views (B and C), for the Uni Adhe.

The interface analysis showed a thicker layer by the presence of the universal adhesive over the grounded zirconia in the Uni Adhe group (Fig. 4A–D). For the other groups, a regular and thinner interface was observed. Regarding the fractographic analysis, all specimens from all evaluated groups failed at the bottom surface of the zirconia (which was faced down during the flexural fatigue test), from a surface defect where the crack initiated and propagated until the fracture of the complete set (ceramic and composite repair) (Fig. 5 A and B). Similarly, the finite element analysis (Fig. 6A) showed a tensile stress concentration at the bottom of the zirconia disc (Fig. 6B). The same pattern was observed for all the evaluated groups (Fig. 6C).

## 4 Discussion

After the grinding protocol by using a diamond bur, all specimens showed similar roughness values. However, the surface topography was modified by the air-abrasion protocols (with and without silica), thus the first null hypothesis was partially rejected. Notable, the impact of the alumina particles of the air-abrasion approaches was able to change the pattern of the defect on the ceramic surface (Fig. 2), thus reducing the scratches of the grinding protocol, which remained present in the Uni Adhe group. These findings are corroborated by a previous study that evaluated the potential of air-abrasion protocols to modify the ceramic surface and affect bonding and mechanical outcomes [34].

Several approaches have been tested by researchers and clinicians to improve the reactivity of zirconia when bonded to the resin composite or resin cement [14,15,17,18]. According to the findings of the present study, the bond strength (static and fatigue) between translucent zirconia and the repair resin composite was affected by the performed surface treatments, thus rejecting the second null hypothesis. Regarding the static test, *Si*-Sil+ and Uni Adhe showed the highest values of bond strength (Table 3), while *Si*-MDP and Air-MDP showed lower values. Silica coating followed by silane application was previously reported as an effective option to promote adhesion to zirconia since the chemical bonding between the incrusted silica particles and resin-based materials is optimized by the action of the bi-functional coupling silane agent, that binds both composite organic content and present silica on the zirconia surface [15,[35], [36], [37]]. In addition, previous studies reported that the use of a universal adhesive can be a suitable alternative to promote sufficient bond strength between zirconia and composite material, even without a pre-treatment [13,36,38], due to its chemical components (MDP), which interacts with the zirconia surface [39]. The MDP component presents a terminal functional group containing phosphoric acid, which reacts with the zirconia surface to form *P*–*O*–Zr bonds, while the other end of the molecule contains a vinyl terminal group, that allows the copolymerization with the resin-based material [15]. Moreover, it is imperative to note that the static shear bond strength test was only carried out under non-aged conditions. Thus, it was necessary to conduct the fatigue shear bond strength test to evaluate the interface stability for each surface treatment in the long term [27].

When considering this fatigue scenario (fatigue shear bond strength test), *Si*-Sil also showed the highest values for fatigue life when compared to the Uni Adhe group. This may be explained by the bonding stability achieved by the silica-coating surface treatment associated with the cumulative effect of silane and MDP in the same primer, which was reported by previous studies [15,40]. The use of primers containing both components is beneficial since air abrasion with silica-coated alumina particles usually leaves untreated areas in the abraded material, leading to a not homogenous and uniform surface. In these areas, the MDP component takes place and acts on the surface, as mentioned [15]. This may also explain the maintenance of the bond strength levels of *Si*-MDP and Air-MDP when compared to the static bond strength test, where no aging protocol was applied. On the other hand, the main chemical bonding promoted by universal adhesive seemed to be not enough stable during the cyclic load application, which probably degraded the adhesive layer and the interface; consequently, decreasing the bond strength between zirconia and the repair resin composite (Fig. 4).

Regarding the fatigued biaxial flexural strength of the repaired 4 YSZ, the silica-coated alumina particles surface treatment associated with MDP-containing silane (*Si*-Sil) depicted the highest fatigue strength, being statistically different from the use of only a universal adhesive containing MDP (Uni Adhe). This may be explained by the association of both chemical and mechanical factors to improve the interlocking between 4 YSZ and resin composite. Therefore, the third hypothesis was rejected. As mentioned, the introduction of silica and the use of an MDP-containing silane (*Si*-Sil) promoted a stronger chemical bond through links with the methacrylate, besides increasing the surface energy and wettability of the composite, thus chemically reinforcing the bonding interface [15,24,36]. Besides, the modified surface topography of zirconia after the air-abrasion with silica-coated alumina particles probably also increased the micro-retention and the interaction with the composite [17,37]. These factors probably allowed a more effective filling of the ceramic surface by the resin composite (Fig. 4) when compared to the Uni Adhe group, generating a reinforcement effect on the set during the mechanical test [41,42], as corroborated by the finite element analysis (Table 3, Fig. 6).

When considering the three strategies where air-abrasion was performed, alumina particles associated with primer 10-MDP (Air-MDP) and silica-coated alumina particles associated with MDP-containing silane (*Si*-Sil) or just primer 10-MDP (*Si*-MDP) presented similar mechanical and adhesive performances. These findings corroborate previous studies that considered the combination of mechanical and chemical approaches as the best choice to increase the performance of zirconia ceramics [15,17]. In addition, based on mechanical behavior, a previous study has demonstrated that surface treatments are crucial for enhancing the fatigue performance of luted monolithic zirconia restorations. The most effective options for achieving high and similar results for such outcomes are air-abrasion with alumina particles and silica-coated treatments [37].

Regarding the failure pattern during the mechanical tests, all fractures originated in the bottom surface of the zirconia specimens (Fig. 5), which was faced down in the machine. This is by the stress concentration depicted by the finite element analysis (Fig. 6), which showed high tensile stress values at that location, thus leading to crack origination and propagation until the catastrophic failure.

Despite the findings of the present study, it must be noted that there are some limitations. Due to a programming constraint during the mechanical tests, it was not possible to generate specific stress parameters (measured in MPa) for each tested specimen. Consequently, fatigue failure load was used to collect the data, and the final values were analyzed using numerical analysis to define the fatigue strength of each group (Table 3). The analysis considered the 10 % mesh convergence for significant differences. Additionally, the thickness of the set was strictly controlled within a range of 0.02 of variability, which ensures that all the obtained data are reliable. However, the *in vitro* evaluation of a fatigue scenario does not include all the factors present in a clinical scenario, such as pH, oscillation of temperature, and different masticatory movements. Thus, even though these findings are essential to show the material behavior in a controlled environment, the obtained findings must be considered with caution. In addition, only one category of resin composite material was used for the repair procedures in this study. Therefore, it is recommended that future studies evaluate different composites, such as bulk-fill materials, flowable resin, or even indirect techniques, to enhance the breadth and depth of the available data.

## 5 Conclusion

When repairing 4 YSZ zirconia surfaces, the association of mechanical and chemical approaches is essential for stable bond strength and optimized mechanical behavior, being air-abrasion protocols and the use of silane and/or MDP-based primers suitable for zirconia repair protocols. The use of a universal adhesive alone proved not to be as efficient as the other alternatives.

## Data availability statement

The data associated with the present study have not been deposited into a publicly available repository. The data will be made available upon request to the first author.

## CRediT authorship contribution statement

**Pablo Machado Soares:** Writing - review & editing, Writing - original draft, Visualization, Validation, Software, Methodology, Investigation, Formal analysis, Data curation, Conceptualization. **Marilia Pivetta Rippe:** Writing - review & editing, Supervision, Resources, Project administration, Investigation, Funding acquisition, Conceptualization. **Lucas Saldanha da Rosa:** Writing - review & editing, Writing - original draft, Validation, Software, Methodology, Investigation, Formal analysis, Conceptualization. **Rafaela Oliveira Pilecco:** Writing - review & editing, Writing - original draft, Visualization, Validation, Software, Methodology, Investigation, Formal analysis, Conceptualization. **Gabriel Kalil Rocha Pereira:** Writing - review & editing, Visualization, Validation, Supervision, Software, Resources, Methodology, Investigation, Funding acquisition, Formal analysis, Conceptualization. **Amanda Maria de Oliveira Dal Piva:** Writing - review & editing, Supervision, Project administration, Investigation. **João Paulo Mendes Tribst:** Writing - review & editing, Visualization, Supervision, Project administration, Methodology, Investigation, Formal analysis. **Luiz Felipe Valandro:** Writing - review & editing, Supervision, Resources, Project administration, Funding acquisition. **Cornelis Johannes Kleverlaan:** Writing - review & editing, Supervision, Resources, Project administration, Investigation, Funding acquisition, Conceptualization.

## Declaration of competing interest

The authors declare that they have no known competing financial interests or personal relationships that could have appeared to influence the work reported in this paper.
